# Flyway‐scale GPS tracking reveals migratory routes and key stopover and non‐breeding locations of lesser yellowlegs

**DOI:** 10.1002/ece3.9495

**Published:** 2022-11-09

**Authors:** Laura A. McDuffie, Katherine S. Christie, Audrey R. Taylor, Erica Nol, Christian Friis, Christopher M. Harwood, Jennie Rausch, Benoit Laliberte, Callie Gesmundo, James R. Wright, James A. Johnson

**Affiliations:** ^1^ U.S. Geological Survey Alaska Science Center Anchorage Alaska USA; ^2^ Alaska Department of Fish and Game, Threatened, Endangered and Diversity Program Anchorage Alaska USA; ^3^ Department of Biological Sciences University of Alaska Anchorage Anchorage Alaska USA; ^4^ Biology Trent University Peterborough Ontario Canada; ^5^ Environment and Climate Change Canada Canadian Wildlife Service Toronto Ontario Canada; ^6^ U.S. Fish and Wildlife Service Kanuti National Wildlife Refuge Fairbanks Alaska USA; ^7^ Environment and Climate Change Canada Canadian Wildlife Service Yellowknife Northwest Territories Canada; ^8^ Environment and Climate Change Canada Wildlife Management and Regulatory Affairs Gatineau Quebec Canada; ^9^ U.S. Fish and Wildlife Service Migratory Bird Program Anchorage Alaska USA; ^10^ School of Environment and Natural Resources The Ohio State University Columbus Ohio USA

**Keywords:** bird migration, lesser yellowlegs, migratory connectivity, migratory route, stopover, *Tringa flavipes*

## Abstract

Many populations of long‐distance migrant shorebirds are declining rapidly. Since the 1970s, the lesser yellowlegs (*Tringa flavipes*) has experienced a pronounced reduction in abundance of ~63%. The potential causes of the species' decline are complex and interrelated. Understanding the timing of migration, seasonal routes, and important stopover and non‐breeding locations used by this species will aid in directing conservation planning to address potential threats. During 2018–2022, we tracked 118 adult lesser yellowlegs using GPS satellite tags deployed on birds from five breeding and two migratory stopover locations spanning the boreal forest of North America from Alaska to Eastern Canada. Our objectives were to identify migratory routes, quantify migratory connectivity, and describe key stopover and non‐breeding locations. We also evaluated predictors of southbound migratory departure date and migration distance. Individuals tagged in Alaska and Central Canada followed similar southbound migratory routes, stopping to refuel in the Prairie Pothole Region of North America, whereas birds tagged in Eastern Canada completed multi‐day transoceanic flights covering distances of >4000 km across the Atlantic between North and South America. Upon reaching their non‐breeding locations, lesser yellowlegs populations overlapped, resulting in weak migratory connectivity. Sex and population origin were significantly associated with the timing of migratory departure from breeding locations, and body mass at the time of GPS‐tag deployment was the best predictor of southbound migratory distance. Our findings suggest that lesser yellowlegs travel long distances and traverse numerous political boundaries each year, and breeding location likely has the greatest influence on migratory routes and therefore the threats birds experience during migration. Further, the species' dependence on wetlands in agricultural landscapes during migration and the non‐breeding period may make them vulnerable to threats related to agricultural practices, such as pesticide exposure.

## Graphical abstract

Since the 1970s, the lesser yellowlegs (*Tringa flavipes*) has experienced a pronounced reduction in abundance of ~63%. The potential causes of the species’ decline are complex and interrelated and understanding the timing of migration and seasonal routes used by this species will aid in directing conservation planning to address potential threats. Our objectives were to identify migratory routes, quantify migratory connectivity and describe key stopover and non‐breeding locations.

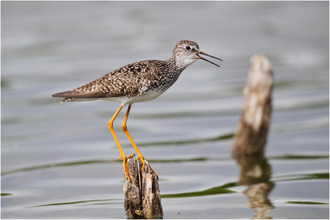

## 1 INTRODUCTION

The Arctic, subarctic, and boreal regions of North America provide habitat for several dozen breeding shorebird species (CHASM, 2006) and nearly all embark on long‐distance migrations to the tropical and temperate habitats of southern latitudes during the non‐breeding season (Myers et al., 1987). In recent decades, many shorebird populations have experienced steep declines (Stroud et al., 2006; Thomas et al., 2006; Wetlands International, 2012), including ~68% of the 52 shorebird species occurring in North America (Rosenberg et al., 2019). Shorebirds are vulnerable to multiple threats throughout the annual cycle because of their long‐distance migrations across various biomes (Piersma et al., 2016; Piersma & Lindström, 2004; Webster et al., 2002) and their reliance on wetland habitats (CHASM, 2006) providing specific food sources (Mathot et al., 2018; Micael & Navedo, 2018).

The causes of North American shorebird declines are complex and include habitat alteration, agrochemical application, urbanization, unregulated harvest, and climate change (Clay et al., 2012; Watts et al., 2015). Some shorebird populations may be more prone to declines due to constraints in their migratory behavior or their geographic distributions (Thomas et al., 2006). For example, the location of stopovers (i.e., locations with abundant food resources) and their relative use during migration may predispose a species or population to a particular threat, leading to an increased risk of decline (Lisovski et al., 2020; Studds et al., 2017). Understanding migratory patterns is an important first step in identifying when and where birds encounter threats and how migratory characteristics (e.g., routes and chronology) may exacerbate population declines and the effectiveness of conservation actions.

The lesser yellowlegs (*Tringa flavipes*) is a long‐distance Nearctic–Neotropical migrant that breeds in the boreal forest of North America. The species has experienced a significant population decline over the past four decades, resulting in a ~63% reduction in abundance (Andres et al., 2012; Bart et al., 2007; Rosenberg et al., 2019). Information gaps exist for this species with respect to migratory routes and timing, stopover and non‐breeding locations, and migratory connectivity (MC). Furthermore, it is unclear if lesser yellowlegs from different breeding populations coalesce during the non‐breeding period and are exposed to the same threats, or if breeding populations remain separate throughout the annual cycle. However, recent findings suggest that lesser yellowlegs breeding in Eastern Canada are more likely to migrate through or to jurisdictions in South America and the Caribbean that practice shorebird harvest than Alaskan and Central Canadian breeding populations (McDuffie et al., 2021).

General patterns of lesser yellowlegs migration routes are known, but until recently, an understanding of individual bird movements from geographically distinct breeding populations was lacking. Abundance estimates of shorebirds along the Atlantic Americas Flyway suggest that lesser yellowlegs breeding in Eastern Canada likely stopover in Atlantic Canada and the east coast of the United States on southbound migration (McNeil & Cadieux, 1972). Much less is known about individuals breeding in Alaska and Central Canada, but count data indicate that lesser yellowlegs are common in the Pacific Northwest during southbound migration (Paulson, 1993). During northbound migration, observational data and a small sample of band recoveries suggest that birds migrate from northern South America, across the Gulf of Mexico, and through the interior plains of the United States (Bent, 1927; Ridgely & Gwynne, 1989; Wunderle et al., 1989). These early reports relied on observations and historic banding and resighting records, which allowed for the description of broad‐scale movement patterns, but not information specific to the annual distributions of individuals from different breeding and post‐breeding origins. Since these accounts, tracking technology has evolved and the migratory movements of this small shorebird can now be characterized, leading to more informed management and conservation decisions (Bridge et al., 2011). A clear understanding of migratory timing, connectivity, and routes is a missing link for lesser yellowlegs (Tibbitts & Moskoff, 2020), and we addressed this knowledge gap by attaching GPS satellite transmitters to adults and following them throughout their annual cycle.

Our objectives were to synthesize high‐resolution data on lesser yellowlegs migratory timing and distance, migratory routes and connectivity, and stopover and non‐breeding locations. Because this information is provided in the form of spatially explicit locations, the results of this study can help identify specific locations where lesser yellowlegs encounter probable threats (e.g., habitat alteration and exposure to agrochemicals). These data can assist in developing conservation and management strategies to mitigate threats and slow or reverse current population declines.

## 2 MATERIALS AND METHODS

### 2.1 Study locations

**FIGURE 1:**
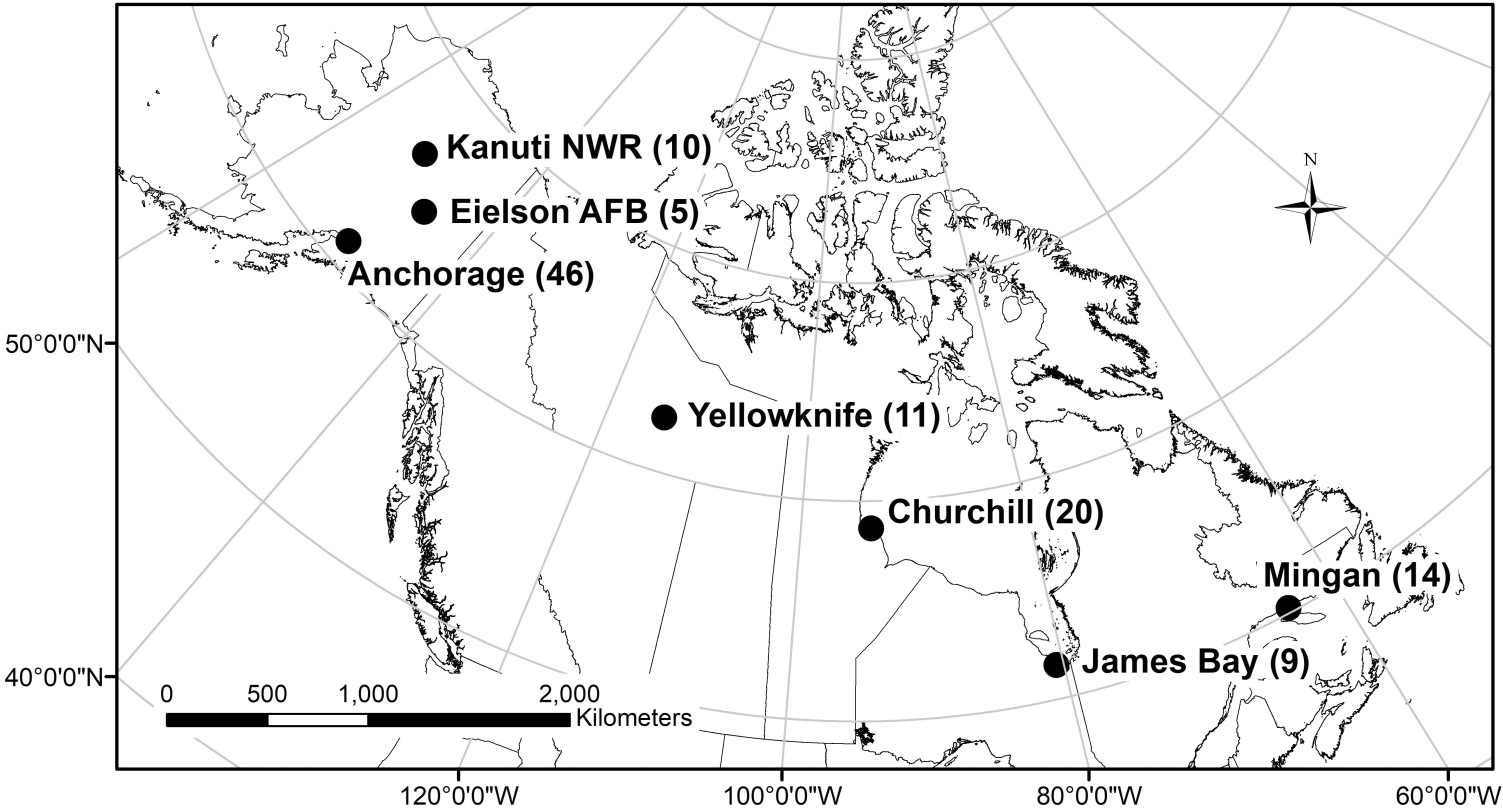
Map of study locations for seven GPS‐tracked populations of lesser yellowlegs. Numbers in parentheses indicate the total number of individual birds with GPS tags per population.

We selected seven locations across the lesser yellowlegs' breeding and early‐migratory distributions for the deployment of GPS transmitters: Anchorage, Alaska, USA (61.2181° N, 149.9003° W); Eielson Air Force Base, USA (AFB; 64.6771° N, 147.0920° W); Kanuti National Wildlife Refuge, Alaska, USA (NWR; 66.4167° N, 151.8333° W); Yellowknife, Northwest Territories, Canada (62.4540° N, 114.3718° W); Churchill, Manitoba, Canada (58.7684° N, 94.1650° W); James Bay, Ontario, Canada (53.5369° N, 80.5457° W); and Mingan Archipelago, Quebec, Canada (50.2205° N, 63.6242° W; Figure 1). We chose these locations based on the availability of logistical support by collaborators and the known presence of lesser yellowlegs (≥5 pairs or 10 adults). We grouped these seven locations into three main populations: Alaska, Central Canada, and Eastern Canada. Anchorage, Kanuti NWR, and Eielson AFB were combined as the “Alaskan population,” Yellowknife and Churchill as the “central Canadian population,” and James Bay and Mingan [archipelago] as the “eastern Canadian population.” The Eastern Canadian population was comprised of birds that breed in Ontario, Quebec, and Labrador and Newfoundland, as determined by GPS tags that transmitted and received for a full annual cycle (*n* = 6).

### 2.2 Field methods and tracking summary

We captured adult lesser yellowlegs in Alaska and Central Canada during the incubation and brood‐rearing periods (May–July). At locations in Eastern Canada, we captured adults during their presumed southbound migration (July–August). At locations in Alaska and Yellowknife, we used mist nets and chick calls (Johnson et al., 2020) to capture brood‐rearing adults. In Churchill, we used shorebird decoys and foraging call recordings to attract lesser yellowlegs. At James Bay and Mingan, we used a combination of mist nets and cannon nets to target foraging and roosting birds. Once captured, we attached to each bird an alphanumeric leg flag, a plastic color band corresponding to the study site, and a USGS metal band. We recorded standard morphometric measurements and collected blood samples to determine sex using molecular markers (Griffiths et al., 2002).

We fit birds with 4.0g PinPoint GPS Argos‐75 satellite tags (hereafter, GPS tag; Lotek Wireless) using a modified leg‐loop harness (Rappole & Tipton, 1991; Sanzenbacher et al., 2000) made of 1.0‐mm‐diameter elastic cord (Stretch Magic, Pepperell Braiding Company) secured in position using 0.5‐mm‐diameter brass jewelry crimps (T.L. Tibbitts, personal communication) and instant adhesive gel (Loctite 454, Henkel Corporation). We selected GPS tags for lesser yellowlegs over other transmitter types because they are lightweight, collect accurate locations (~10 m), and transmit data remotely (Clements et al., 2022). The cumulative weight of the auxiliary leg bands and GPS tags including harness material was ~5.0 g. We only deployed GPS tags on birds weighing a minimum of 80 g [87.68 ± 6.77 *SD* g] to follow the weight restriction guidelines of auxiliary markers (i.e., markers ≤5% body mass) approved by the USGS Bird Banding Lab, The Canadian Bird Banding Office, the University of Alaska Anchorage (UAA), U.S. Fish and Wildlife Service (USFWS), and Alaska Department of Fish and Game (ADF&G) Institutional Animal Care and Use Committees (IACUC; see Ethics Statement).

### 2.3 Tag schedules and processing

We enabled GPS tags to receive and transmit data for the complete annual cycle by selecting schedules that would maximize the amount of data collected while accounting for the estimated battery life of each GPS tag (Table S1). The data received by GPS tags via the Argos system (CLS America, Inc.) were filtered using the Lotek Argos‐GPS Data Processor (Lotek Wireless Inc., v4.2). For our analyses, we only used GPS locations with 2D or 3D fixes (±10 m accuracy; Clements et al., 2022), which refers to the number of messages received from the Argos satellites. Locations categorized as 3D are derived from ≥4 messages, whereas 2D are derived from ≥3 messages (CLS America, 2016). Additionally, we only used locations that passed automated cyclic redundancy checks, which detect invalid changes to raw data (e.g., incorrect removal of the negative sign from the longitudinal position).

### 2.4 Seasonal periods

Seasonal periods included migration, stopovers, and non‐breeding locations of individuals (Table 1). Movements of lesser yellowlegs during the non‐breeding period varied among individuals, with some birds remaining relatively sedentary within a small region (i.e., one‐degree latitude by one‐degree longitude) throughout the non‐breeding period, while others continuously traveling between locations during the non‐breeding period. For individuals that did not move >50 km between GPS locations during the full non‐breeding period, the non‐breeding location was defined as the geographic median of all GPS points received upon arrival to the location and prior to the onset of northbound migration. For individuals that continuously moved throughout the non‐breeding period, we defined the non‐breeding location as the geographic median of the southernmost cluster of GPS points that were <50 km apart. These non‐breeding locations were used in analyses of migratory distance and connectivity, but not migratory duration because data gaps >14 days were prevalent during the non‐breeding season. Non‐breeding locations could only be determined for birds whose GPS tag continued to transmit data through 23 November. We chose 23 November because this date marks the beginning of the non‐breeding period when large‐scale movements of lesser yellowlegs are less frequent, based on relative abundance estimates from the species account in “The Birds of the World” (Tibbitts & Moskoff, 2020) and records in eBird (Fink et al., 2020).

**TABLE 1:** Definitions for seasonal periods and dates of GPS‐tracked lesser yellowlegs between 2018 and 2022

Periods and dates	Definition
Southbound migration	The period between departure from the breeding site and arrival at the terminal non‐breeding location
Northbound migration	The period between departure from the non‐breeding location and arrival at the breeding location
Non‐breeding	The period between the termination of southbound migration and the commencement of northbound migration
Stopover	A location with adequate food resources and environmental conditions where a bird stops migrating for ≥2 days and travels <50 km between any two locations
Breeding departure date	Date of a bird's last occurrence at a breeding location prior to traveling in a unidirectional trajectory of >100 km

### 2.5 Statistical analyses

The GPS tags used in this study recorded locations at intervals >24 h (Table S1). Therefore, we used *foieGras* (version 0.4.0) in program R (R Core Team, 2022) to interpolate data gaps in migratory routes by generating a continuous state–space random walk model for each individual bird (Jonsen et al., 2019). A random walk model portrays stochastic movements that are uncorrelated and unbiased (Codling et al., 2008). The *foieGras* model uses this principle to estimate locations that correspond to real observations transmitted by each GPS tag. This model requires that location data include the following parameters: location class, semi‐major and semi‐minor axis of an ellipse, and an ellipse error value. The location class indicates the total number of messages received per satellite pass and the location accuracy. The error ellipse indicates the estimated distance (i.e., semi‐major and semi‐minor axes) that the estimated location is from the actual location. All the location data used in the *foieGras* model were GPS derived; therefore, the location class was set as “3D” (i.e., indicating that the data are GPS and not Doppler derived). Additionally, because the error around GPS locations is considered minimal, we used an error ellipse of 0 and 300 m as the semi‐major and semi‐minor axis values, respectively (Douglas et al., 2012). Next, we ran the location data through a pre‐filter and fit the model to estimate a location every 24 h using an estimated flight velocity of 15 m/s (54 km/h; Pennycuick et al., 2013). Finally, we fit the pre‐filtered model to the projected version of the data. Using the random walk model, we estimated the locations of traveling birds throughout southbound and northbound migrations; however, the model began to degrade for data gaps >4 days (96 h).

#### 2.5.1 Breeding departure dates and migratory distance

We used the last true or estimated date (i.e., if data gaps were <4 days) a bird was present at the breeding location as the southbound departure dates for males and females. To test if the departure date from breeding locations depends on the sex of a bird and breeding population from which a bird originated, we computed a two‐way ANOVA with an interaction effect (R Core Team, 2022). Only individuals breeding in Anchorage, Kanuti NWR, Yellowknife, and Churchill could be used in the analysis. Birds tagged at Eielson AFB in Alaska were not sexed and data gaps of 7 days (168 h) existed for the first fix interval period (Table S1), which caused the *foieGras* estimation model to deteriorate. Therefore, we removed this site from breeding departure or migratory distance analyses. The James Bay and Mingan lesser yellowlegs were also removed because these birds were tagged during southbound migration.

We used the Geographic Distance Matrix Generator v1.2.3 (Ersts, 2007) to calculate migratory distance. The application assumes that the Earth is a perfect sphere and uses the semi‐major axis of the WGS84 reference system as the default radius of the Earth. We calculated the southbound migratory distances as the cumulative distance a bird traveled (km). This distance is not a unidirectional straight line but includes all intermediate distances between consecutive locations throughout migratory periods, including omnidirectional movements at stopover locations.

Lastly, to identify which factors predicted migratory distance, we used a generalized linear model assuming a negative binomial error distribution and a log‐link function using package *MASS* in Program R, version 7.3‐58 (R Core Team, 2022; Venables & Ripley, 2002). Covariates of the model included sex, capture mass, the longitude of capture location, and departure date (i.e., Julian date) from the breeding location. We used Pearson's product–moment correlation test to assess if correlations between covariates existed, and when high correlations (*r* > .60) were identified, we used Akaike's information criterion corrected for small sample sizes (AIC_c_; Burnham & Anderson, 2002) to select the covariate that best fit the data. Additionally, we compared the fit of negative binomial and Poisson distribution models and determined that the negative binomial model was the best fit for the available data. Models including all possible additive combinations of non‐correlated covariates were compared using AIC_c_. We did not conduct this analysis for northbound migration due to limited sample size and incomplete data on departure dates and body mass. Furthermore, James Bay and Mingan birds were excluded because they were captured during southbound migration.

#### 2.5.2 Migratory routes and connectivity

We visualized the migration routes of individuals by plotting GPS locations and *foieGras* estimated model locations using the Mercator projection in ArcMap 10.6 (ESRI, 2018). Track lines between consecutive locations were plotted using the “XY to lines” tool, which created a geodesic line that most accurately represents the shortest distance between two points on the Earth's surface. Next, we divided track lines into southbound and northbound migration based on the definitions of migratory seasons (Table 1).

Migratory connectivity refers to the correlation of distances between individuals at two different locations, such as breeding and non‐breeding locations. It can range from weak, when individuals from different breeding populations mix on the non‐breeding grounds, to strong, when there is segregation of breeding populations (Cohen et al., 2018; Webster et al., 2002). An MC value of zero indicates weak connectivity and no relationship in distances between two periods, whereas MC values close to 1 indicate strong connectivity (Cohen et al., 2018). We calculated MC using the *estMC* function in the *MigConnectivity* package (version 0.4.1; Hostetler & Hallworth, 2018) in program R (R Core Team, 2022) with 10,000 bootstrap samples and 10,000 simulations.

The *estMC* function requires the input of predefined origin and target locations. We used the three inclusive populations (i.e., Alaskan, Central Canadian, and Eastern Canadian) to determine the strength of MC. We defined the geographic median capture locations as the origins of each population. Next, we defined target locations by level 1 ecoregion type (CEC, 1997; Griffith et al., 1998; Lawler et al., 2009). Therefore, the MC analysis included 3 origin locations (populations) and 10 target locations (non‐breeding): Mediterranean California (California), Temperate Sierras (Mexico), Tropical Dry Forests (Mexico), Tropical Wet Forests (Mexico), West Indies (The Caribbean), Northern Andes (Venezuela and Colombia), Amazonian‐Orinocan Lowland (Brazil), Central Andes (Ecuador and Peru), Gran Chaco (Argentina and Bolivia), and Pampas (Argentina and Uruguay). The *estMC* function also incorporates relative abundance, transition probabilities, and geographic uncertainty into the model. We used the estimated abundance of lesser yellowlegs within different Bird Conservation Regions (BCR) of North America as a measure of relative abundance (Partners in Flight, 2021). The transition probabilities included the proportion of birds per origin occurring within a particular ecoregion (i.e., target locations) during the non‐breeding period. The analysis included both 2D and 3D Argos locations and we used zero as the spatial uncertainty because the accuracy of GPS tags for GPS (i.e., 2D and 3D locations) is approximately 10 m (Clements et al., 2022).

#### 2.5.3 Stopover and non‐breeding locations

We identified the relative importance of stopover and non‐breeding locations using the number of unique individuals that stopped within a geographic area. We defined stopover locations based on the duration of stay and distance traveled between consecutive locations (Table 1; Warnock, 2010). Stopover and non‐breeding locations were composed of multiple locations per individual; therefore, we used the “median center” tool in ArcMap (ESRI, 2018) to calculate a single geographic median location for each bird.

## 3 RESULTS

We deployed 118 GPS tags on adult lesser yellowlegs (52 males, 60 females, and 6 of unknown sex; Table S2). Variability in GPS‐tag schedules across years, intermediate lapses in satellite communication, and the presumed mortality of some birds resulted in the partial loss of functional data during migratory periods. Fifty‐two GPS tags provided data on breeding departure dates and full migratory track lines through the non‐breeding period, which is required to determine southbound migratory distance. One hundred and fifteen tags provided full or partial information on migratory routes, while the remaining three GPS tags failed to transmit/receive at all. Sixty‐eight tags were used to calculate MC because these tags transmitted and received locations through November 23 each year (see “Section 2.4” for a description of migratory seasons). Finally, 90 tags were used for stopover and non‐breeding location determination because 28 tags failed to transmit/receive prior to the first observed stopover.

### 3.1 Breeding departure dates and migratory distance

**FIGURE 2:**
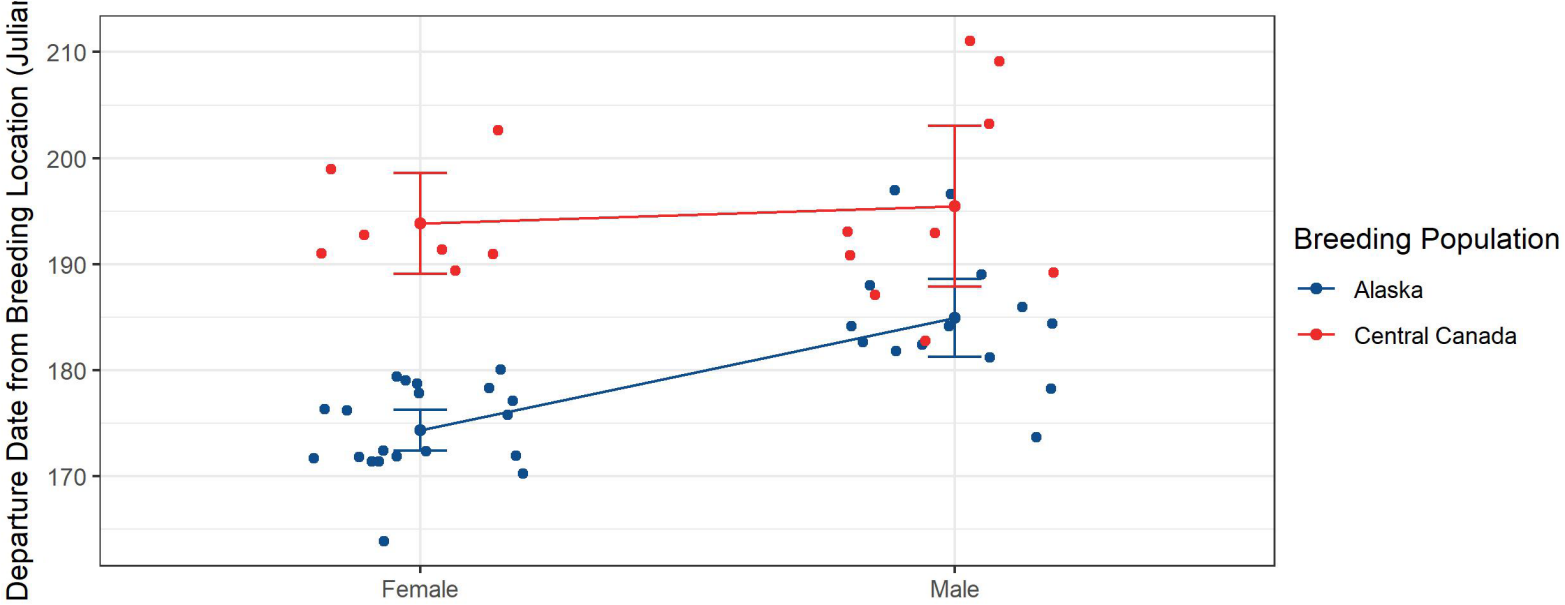
Line plot showing the relationship of departure date from breeding locations (Julian date), breeding population (Alaskan and Central Canadian), and sex. Whisker plots represent the mean (center point) and 95% confidence intervals. Alaskan population (*n* = 34; 20 females and 14 males) and Central Canadian population (*n* = 16; 7 females and 9 males). Departure dates are in Julian days (e.g., 170 = June 19 and 210 = July 29).

Departure dates from breeding locations were earlier for Alaskan than Central Canadian populations. Also, females departed earlier than males for Alaska, while departure dates were similar for males and females breeding in Central Canada (Figure 2). The effect of sex and breeding population were both significantly associated with departure date [sex = 0.05 (−0.01, 0.11); breeding population = 0.06 (0.01, 0.11)] with a moderate‐effect size (Cohen's *d* = 0.35).

**FIGURE 3:**
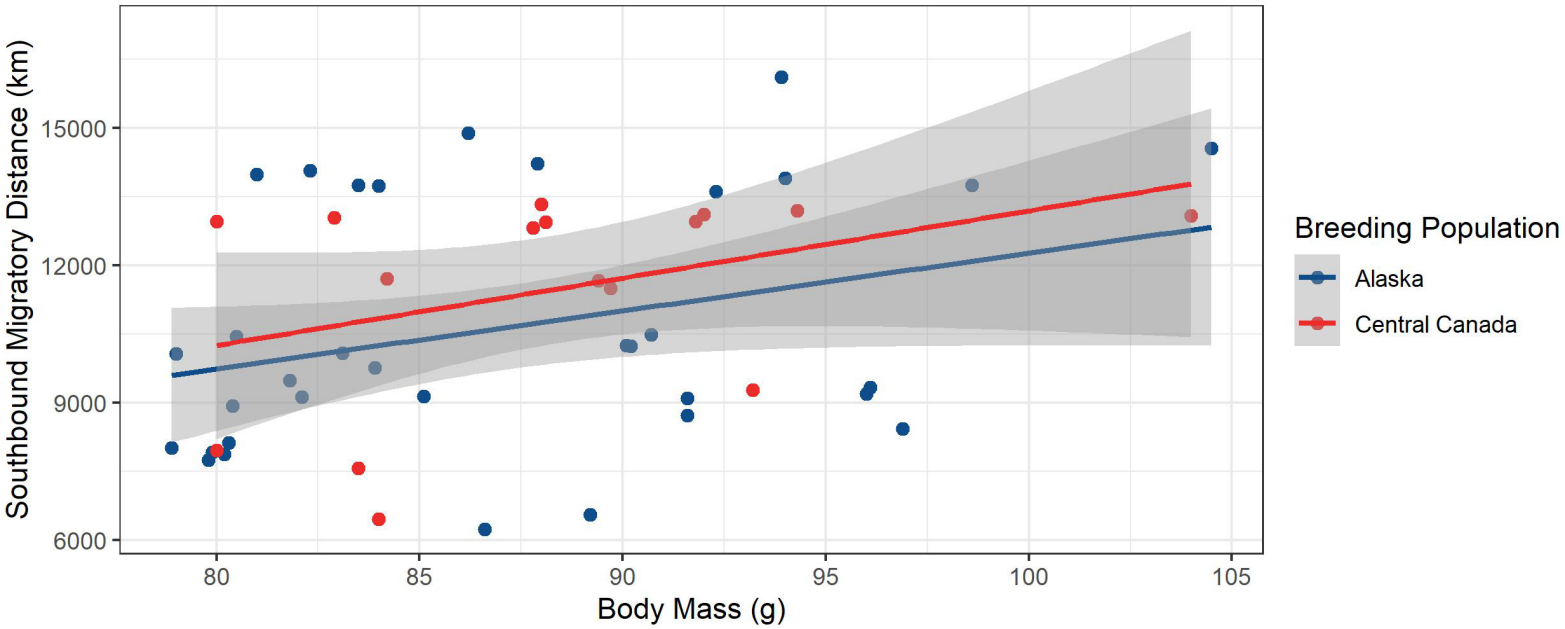
Scatter plot showing the relationship of southbound migratory distance (km) and body mass (g) of lesser yellowlegs from Alaskan and Central Canadian populations. The blue and red lines represent the fitted linear regression lines for Alaska and Central Canada and the gray bands represent the 95% confidence interval bands. Alaskan population (*n* = 34; 20 females and 14 males) and Central Canadian population (*n* = 16; 7 females and 9 males).

For the analysis of factors affecting migratory distances, there was a strong correlation between the covariate's departure date and capture longitude (Pearson's *r* = .72). Capture longitude was included and the departure date was excluded for subsequent models based on the AICc selection criteria. Body mass and sex were moderately correlated (Pearson's *r* = .56), and therefore, both covariates remained in the models. The top‐ranked model included the single covariate body mass at time of capture (AIC weight = 0.40). The top two models were similar (Δ*i* = 1.68) indicating that there is some evidence supporting the second‐ranked model (Burnham & Anderson, 2002; Table 2), which included body mass at time of capture and capture longitude (AIC weight = 0.57). However, 95% confidence intervals for the capture longitude estimate overlapped zero; therefore, the only clear predictor of migratory distance was body mass, with heavier birds migrating greater distances (Figure 3).

**TABLE 2:** Relationship between migratory distance and spatial and biological covariates: Sex, capture mass, and capture (GPS deployment) longitude. Models were fit using a binomial generalized linear model and Akaike's information criterion for model selection.

Model	*k*	Parameters^b^				*W*_*i*_	ΔAIC
		Intercept	Sex Male	Capture mass	Capture longitude		
D (capture mass)	3	8.2121 (7.35, 9.08)	NA	0.0124 (0.00, 0.02)	NA	0.40	0.00
H (capture mass + capture longitude)	4	8.4015 (7.43, 9.37)	NA	0.0118 (0.00, 0.02)	−0.0011 (−0.00, 0.01)	0.57	1.68
F (sex + capture mass)	4	8.3261 (7.24, 9.42)	−0.0263 (−0.18, 0.13)	0.0112 (−0.00, 0.02)	NA	0.69	2.25
C (sex)	3	9.3457 (9.26, 9.43)	−0.1082 (−0.24, 0.02)	NA	NA	0.78	3.15
B (intercept only)	2	9.2973 (9.23, 9.36)	NA	NA	NA	0.85	3.43
A (sex + capture mass + capture longitude)^a^	5	8.6057 (7.38, 9.83)	−0.0414 (−0.19, 0.11)	0.0099 (−0.00, 0.02)	0.0012 (−0.00, 0.00)	0.90	3.89
G (sex + capture longitude)	4	9.5739 (9.21, 9.94)	−0.1171 (−0.25, 0.01)	NA	0.0017 (−0.00, 0.00)	0.96	3.93
E (capture longitude)	3	9.4887 (9.13, 9.85)	NA	NA	0.0014 (−0.00, 0.00)	1.00	4.59

*Note*: *k*, the number of parameters; *w*_*i*_, AIC_c_ weight; ΔAIC, delta AIC, NA, not applicable.

^a^ Model A is the full model and model B is the intercept‐only model.

^b^ Parameter estimates and the lower and upper 95% confidence intervals are in parentheses.

### 3.2 Migratory routes and connectivity

**FIGURE 4:**
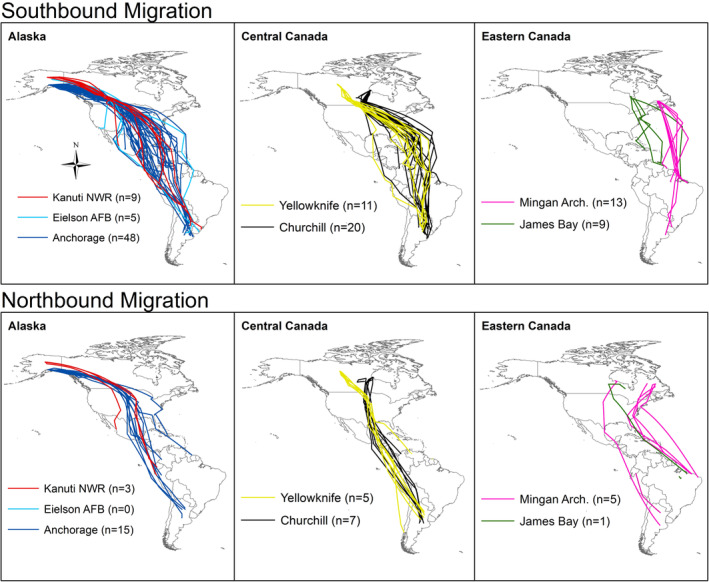
Migratory routes of tracked lesser yellowlegs during 2018–2022. Each track line represents the southbound and northbound movements of an individual bird tagged in Alaska, Central Canada, or Eastern Canada. Southbound migration (*n* = 115; Kanuti NWR = 9, Eielson AFB = 5, Anchorage = 48, Yellowknife = 11, Churchill = 20, Mingan archipelago = 13, and James Bay = 9). Northbound migration (n = 36; Kanuti NWR = 3, Eielson AFB = 0, Anchorage = 15, Yellowknife = 5, Churchill = 7, Mingan archipelago = 5, and James Bay = 1).

Adult lesser yellowlegs originating in Alaska and Central Canada followed similar migratory routes through the Midcontinent Americas Flyways during southbound and northbound migration, while birds migrating from Eastern Canada migrated along the Atlantic Americas Flyway during southbound migration and the Midcontinent Americas and Atlantic Americas Flyways during northbound migration (Figure 4). Migratory routes were similar for birds traveling from similar geographic regions (i.e., Alaska or Central Canada); however, some variation in routes was observed. For example, during southbound migration in 2018, a bird migrating from Anchorage used the Atlantic Americas Flyway to reach a non‐breeding location in Suriname. Further, in 2019, two lesser yellowlegs migrating from Yellowknife and Churchill, respectively, traveled through Central America to reach a non‐breeding location in South America rather than migrating across the Atlantic Ocean (Figure 4).

**FIGURE 5:**
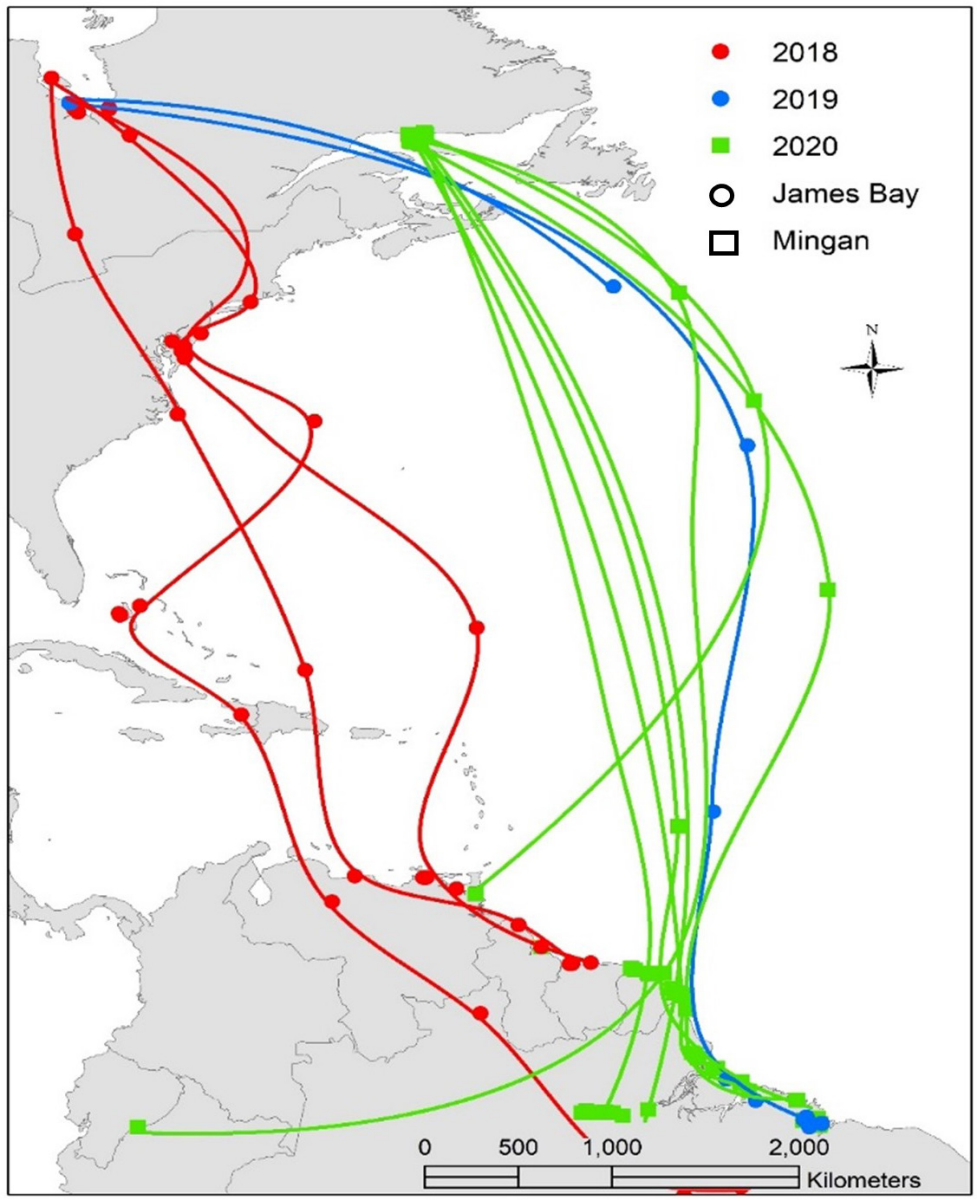
Map showing multi‐day transoceanic migratory routes of the Eastern Canadian population of lesser yellowlegs during 2018–2020. Each track line represents an individual lesser yellowlegs (James Bay = 5 and Mingan = 7).

Several lesser yellowlegs in our study undertook multi‐day transoceanic flights from James Bay and Mingan directly to coastal South America (Figure 5). Ten birds traveled a mean distance of 4500 km across the Atlantic, taking approximately 4 days to reach land. During southbound migration in 2018, individuals from James Bay (*n* = 3) stopped at locations along the Atlantic coastline prior to making a transoceanic flight. In 2019, one tagged bird did not stop along the coast before completing a multi‐day (4.8 ± 1.1 days) transoceanic flight to the Caribbean (Figure 5). All birds tagged in Mingan in 2020 (*n* = 6) completed transoceanic flights to South America (4.7 ± 0.43 days; Figure 5).

The 68 individuals tracked to non‐breeding locations from different breeding origins exhibited weak migratory connectivity (mean MC = 0.174 ± 0 *SE*). Our calculation of MC yielded a standard error of zero because the GPS locations used in the *estMC* function are highly precise. This connectivity value suggests that mixing among populations occurs during the non‐breeding period.

### 3.3 Stopover and non‐breeding locations

**FIGURE 6:**
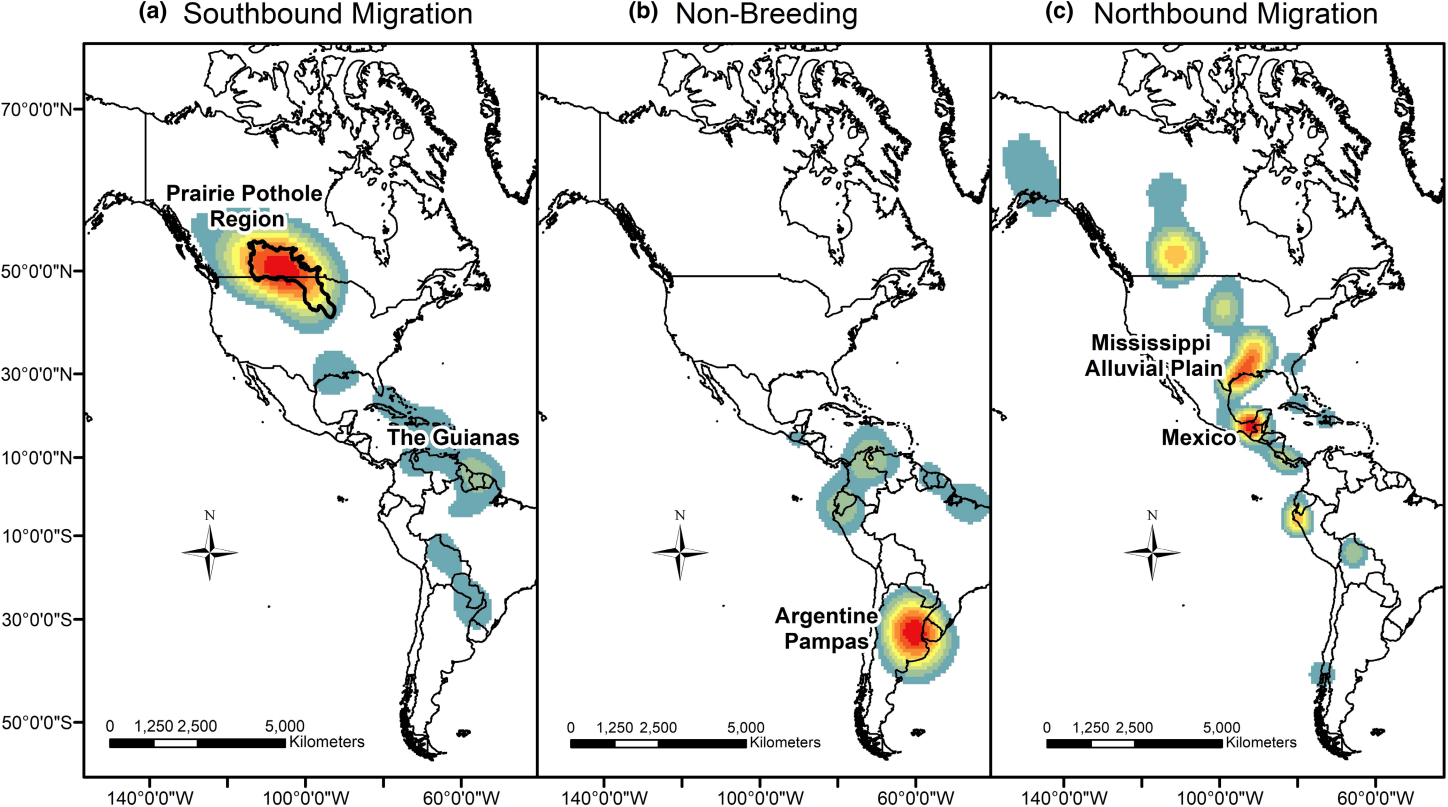
Heatmap showing the density of stopped lesser yellowlegs from seven tracked populations during (a) southbound migration, (b) non‐breeding, and (c) northbound migration. Warm colors indicate locations with the highest density of individuals. The Prairie Pothole Region comprises 33% of stopover locations during southbound migration. Southbound migration includes 89 unique individuals (Alaska = 47; Central Canada = 30; Eastern Canada = 12) and 294 stopover locations (Alaska = 160; Central Canada = 104; Eastern Canada = 30). The Argentine Pampas comprises 26% of locations during the non‐breeding period. The nonbreeding period includes 68 unique individuals and locations (Alaska = 41; Central Canada = 21; Eastern Canada = 6). Mexico and the Mississippi Alluvial Plain comprise 37% of stopover locations during northbound migration. Northbound migration includes 30 unique individuals (Alaska = 18; Central Canada = 11; Eastern Canada = 1) and 46 locations (Alaska = 29; Central Canada = 16; Eastern Canada = 1).

The Prairie Pothole Region (PPR) supported lesser yellowlegs migrating from Alaska (100% of birds), Yellowknife (100%), and Churchill (65%) during southbound migration (Figure 6a). Birds breeding in Alaska and Central Canada mixed within the region during southbound and northbound migration, respectively. The birds from the Eastern Canada populations migrated exclusively along the Eastern United States coastline and across the Atlantic Ocean between North and South America and did not occur in the PPR.

The 1.2 million‐square‐kilometer Argentine Pampas Region encompassing six Argentine provinces, all of Uruguay, and the southernmost state of Brazil, supported the greatest number of non‐breeding lesser yellowlegs (44%), followed by coastal Venezuela (9%), Brazil (9%), and Ecuador (7%; Figure 6b). Of the birds tracked to a non‐breeding location, 40% of birds originating in Alaska or Central Canada spent the non‐breeding period in the Argentine Pampas.

During northbound migration, GPS tracked lesser yellowlegs stopped within a few discrete locations. The Mississippi Alluvial Plain (i.e., spanning the Mississippi River floodplain from Southern Louisiana to Southern Illinois) supported the highest number of individuals (Figure 6c). Of the 36 birds tracked during northbound migration, 25% stopped in the Mississippi Alluvial Plain, 22% in Mexico, and 11% in the PPR.

## 4 DISCUSSION

Through the use of miniaturized GPS tracking technology, we found that lesser yellowlegs' departure timing from breeding locations corresponded to sex and breeding population, such that birds originating in Alaska departed earlier than those originating in Central Canada and females from Alaska departed earlier than males. Total migratory distance did not vary as a function of sex, but we observed a pronounced effect of body mass, with heavier individuals migrating greater distances. Finally, MC was weak with substantial mixing of birds from different breeding origins during the non‐breeding season.

Females departed breeding locations earlier than males in Alaska, which is consistent with the observation that male lesser yellowlegs typically remain with broods longer than females (Tibbitts & Moskoff, 2020). This later departure from breeding locations potentially influences a males' ability to successfully migrate. For example, the depletion in prey abundance during the temporal progression of southbound migration has been documented in shorebird foraging areas in North America and this has the potential to induce energy deficits in late‐arriving birds (Schneider & Harrington, 1981). Also, birds departing breeding locations later must not only align migration phenology with prey availability (Colwell & Landrum, 1993; Newton, 2007) but also avoid or minimize overlap with the migrations of predators. Many Arctic‐breeding shorebirds migrate in July and August, prior to the migration of hawks and falcons, but a delay in migration (e.g., late hatch and prolonged brood care) could put male and juvenile shorebirds at risk of aligning their migration with avian predators (Lank et al., 2003). Local environmental conditions may also influence departure dates and how they differ among sexes. Post‐breeding shorebirds in the subarctic are known to select tailwinds that support migratory efficiency (Duijins et al., 2019). Due to the unique seasonal oceanic and atmospheric conditions in the Hudson Bay Region (Ridenour et al., 2019), birds departing Churchill in late summer may experience more variable wind patterns compared to the Alaskan population, potentially minimizing the window of favorable wind conditions and requiring sexes to depart at similar times. This concept, however, requires further investigation.

Body mass at time of capture was the best predictor of total southbound migratory distance. Birds were captured at the end of incubation and early brood rearing, suggesting that measured body masses represented the minimum potential body condition of each bird prior to southbound migration. The body size hypothesis argues that heavier birds can survive longer periods of fasting (Duijns et al., 2017; Ketterson & Nolan, 1976); therefore, light birds in poor condition may stop earlier to replenish reserves than birds in good condition. Fueling rates in shorebirds can vary across latitudinal gradients (Reneerkens et al., 2020). In the Northern and Southern Hemispheres, fuel‐loading rates (i.e., feed intake) decrease from high to low latitudes (Aharon‐Rotman et al., 2016; Piersma et al., 2005; Williams et al., 2007), suggesting that lesser yellowlegs that migrate to more southern latitudes of South America may be better able to replenish fat reserves. Additionally, foraging rates can be constrained in tropical regions near the equator due to physiological stress from high ambient temperatures (Battley et al., 2003; Speakman & Król, 2010). Tropical non‐breeding regions have also been linked to low adult survival of Arctic‐breeding shorebirds (Reneerkens et al., 2020).

Despite differences in departure timing and migratory distance, we found that Alaskan and Central Canadian populations of lesser yellowlegs followed similar migratory routes during southbound migration, while Eastern Canadian populations migrated further east. The southbound migratory routes used by the different populations corroborate the concept of longitudinal parallel migration (Newton, 2007), where birds breeding in the west tend to migrate farther west than birds breeding in the east. However, during northbound migration, we observed an overlapping pattern where individuals from breeding populations in Alaska and Central Canada followed a similar route within the Midcontinent Americas Flyway, whereas individuals tracked from Eastern Canada followed both the Midcontinent Americas and Atlantic Americas Flyways. There is evidence of overlapping northbound migratory routes in other Neotropical migrants (e.g., breeding populations of Semipalmated Sandpiper, *Calidris pusilla*; Brown et al., 2017).

In addition to the longitudinally parallel migration routes observed during southbound migration, we found evidence indicating that lesser yellowlegs migrating from Eastern Canada complete multi‐day, non‐stop transoceanic flights of >4000 km. This extends our knowledge of shorebird species that are capable of transoceanic flights to those weighing <100 g. Typically, large shorebirds averaging ≥200 g, such as bar‐tailed godwit (*Limosa lapponica*; Battley et al., 2012; Gill et al., 2009) and bristle‐thighed curlew (*Numenius tahitiensis*; Marks & Redmond, 1994) are considered capable of extended non‐stop flights of >4000 km in distance. However, recent studies have indicated that medium‐ and light‐weight shorebirds such as upland sandpiper (*Bartramia longicauda*; mean body mass of 142 g; Hill et al., 2019) and sanderling (*Calidris alba*; mean body mass of 70 g; Conklin et al., 2017) are also capable of transoceanic flights without the need to refuel. Additionally, smaller migrants such as pectoral sandpiper (*Calidris melanotos*; mean body mass of 79 g) and white‐rumped sandpiper (*Calidris fuscicollis*; mean body mass of 42 g) that travel through the James Bay Region are known to complete long‐distance migrations without apparent refueling stops in the United States (Anderson et al., 2019). Finally, a smaller Tringa species, the solitary sandpiper (*Tringa solitaria*; mean body mass of 48 g) has been observed completing transoceanic migrations of several 100 km (Sorte & Fink, 2017).

Although Alaskan and Central Canadian breeding populations of lesser yellowlegs used different southbound migratory paths than Eastern Canadian migratory populations, mixing of populations occurred within non‐breeding locations, resulting in weak MC. Species with weak MC and geographically expansive non‐breeding ranges may be less susceptible to population declines than birds with strong MC and restricted non‐breeding ranges (Gilroy et al., 2016). For example, species with strong MC may be restricted to few non‐breeding locations and may therefore be more susceptible to habitat loss or degradation of those locations (Dolman & Sutherland, 1995). Additionally, populations that experience weak MC may have considerable genetic variation in migratory behavior, which may facilitate adaptation to environmental changes (Webster & Marra, 2005). Yet despite the possible benefits conveyed on lesser yellowlegs by weak MC, the species is declining, suggesting that threats are both pervasive and acute to overpower any benefits associated with weak connectivity. Also, the dependence on certain regions (e.g., Prairie Potholes, Argentine Pampas, and Mississippi Alluvial Plain) by a large proportion of tagged individuals highlights the species' vulnerability to threats in these regions.

Our tracking data indicated that the PPR of North America, Argentine Pampas, and the Mississippi Alluvial Plain are frequent stopover and non‐breeding locations for lesser yellowlegs. The PPR consists of thousands of small wetlands, ponds, lakes, and floodplains surrounded by crop fields that provide foraging habitat for refueling migratory lesser yellowlegs. Between 1997 and 2009, 39% of emergent wetland loss in the PPR of the United States was attributed to agricultural conversion, and of the remaining wetlands, 94% were located adjacent to, or within, crops or pasturelands (Dahl, 2014; Muhammad et al., 2018). Like the PPR, the Argentine Pampas comprises expansive grasslands, pasture, and agricultural land surrounded by wetlands, floodplains, and tributaries. The recent increased demand for soybean exports has resulted in the conversion of free‐range cattle pasture and wetlands to cropland and concentrated cattle feedlots (Rossi, 2015). The Mississippi Alluvial Plain (MAP) in the United States encompasses swamps, bayous, and hundreds of kilometers of rivers. Historically, the region consisted of 9 million hectares of wetland forests, but today only less than a quarter of forests remain following the construction of flood‐control levees and the conversion to agricultural lands (Hoyle, 2015). The MAP region yields the highest returns for aquaculture (i.e., catfish) and rice in the United States (U.S. Department of Agriculture National Agricultural Statistics Service, 2019) and helps drive the regional economy (Alhassan et al., 2019).

The effects of agricultural practices on lesser yellowlegs are a concern across the migratory and non‐breeding range of the species and warrant further investigation. In the PPR, agricultural pesticides, herbicides, and fungicides are applied in large quantities and are known to accumulate in wetlands (Goldsborough & Crumpton, 1998; Malaj et al., 2020; McMurry et al., 2016). Similar agrochemicals are used in the highly agricultural Pampas Region of Argentina (De Gerónimo et al., 2014; Etchegoyen et al., 2017). Furthermore, at Mar Chiquita on the northern edge of the Argentine Pampas, organochlorine pesticides and polychlorinated biphenyls used until 1998 and 2005, respectively, have lingering effects on aquatic habitats (Ballesteros et al., 2014). At the mouth of the Mississippi River, pesticide accumulation from agricultural production in the Mississippi Alluvial Plain results in annual hypoxic conditions (Rabalais et al., 2002) and harmful algae bloom in coastal salt marshes, in turn adversely affecting the food web (Ning et al., 2015) and shorebird foraging behavior (Kvitek & Bretz, 2005).

Habitat conversion to agriculture and, consequently, pesticide application are prevalent in regions that support high densities of lesser yellowlegs. Whether in freshwater systems in the PPR and Argentine Pampas, or marine‐influenced systems at the mouth of the Mississippi River, further investigations are needed to diagnose contaminant loads carried by the species and the effects of contaminants and habitat loss on productivity and survival.

## 5 CONCLUSION

The lesser yellowlegs population is in decline and understanding their migratory ecology is the crucial first step in evaluating the species’ vulnerability to potential threats. Our study suggests that lesser yellowlegs from disparate breeding populations are vulnerable to similar threats throughout migration and the non‐breeding period in relation to migratory pathways and connectivity.

By identifying the distributions of several lesser yellowlegs populations using GPS‐tracking technology, we can start to identify specific regions where more information could be sought to inform conservation actions. For example, working with agricultural experts in understanding possible contaminants and any effects on the productivity and survival of lesser yellowlegs would increase our collective knowledge regarding the effect of agricultural practices on refueling shorebirds. Additionally, future studies could use GPS tags with more frequent fixes to determine more precise migration phenology, including stopover durations and hence key staging locations for targeted conservation efforts.

Further, because our findings indicate that lesser yellowlegs from the same geographic regions follow similar migratory pathways and mix during the non‐breeding period, we suggest assessing genetic structure to determine whether subpopulations exist, despite weak MC. This may help in evaluating the benefit of different future management actions, such as those focused on either the entire lesser yellowlegs population or on individual subpopulations with regard to exposure to prominent threats.

## AUTHOR CONTRIBUTIONS

**Laura A. McDuffie:** Conceptualization (lead); data curation (lead); formal analysis (lead); funding acquisition (equal); investigation (equal); methodology (equal); project administration (equal); resources (equal); supervision (equal); validation (lead); visualization (lead); writing – original draft (lead); writing – review and editing (lead). **Katherine S. Christie:** Conceptualization (lead); funding acquisition (lead); methodology (supporting); project administration (supporting); writing – original draft (supporting); writing – review and editing (supporting). **Audrey R. Taylor:** Conceptualization (equal); funding acquisition (supporting); methodology (supporting); writing – original draft (supporting); writing – review and editing (supporting). **Erica Nol:** Funding acquisition (supporting); project administration (supporting); writing – original draft (supporting); writing – review and editing (supporting). **Christian Friis:** Funding acquisition (supporting); writing – review and editing (supporting). **Christopher M. Harwood:** Project administration (supporting); writing – review and editing (supporting). **Jennie Rausch:** Project administration (supporting); writing – review and editing (supporting). **Benoit Laliberte:** Methodology (supporting); writing – review and editing (supporting). **Callie Gesmundo:** Methodology (supporting); writing – review and editing (supporting). **James R. Wright:** Writing – review and editing (supporting). **James A. Johnson:** Conceptualization (lead); funding acquisition (lead); methodology (lead); project administration (lead); writing – original draft (supporting); writing – review and editing (supporting).

## FUNDING INFORMATION

This study was supported by the 673 CES/CEIEC, U.S. Department of the Air Force (project numbers FXSB46058118, FXSB4658119, and FXSBA53216120) which required approval of this manuscript before publication, Alaska Department of Fish and Game, Birds Canada, Environment and Climate Change Canada (Canadian Wildlife Service), Parks Canada (Mingan), U.S. Fish and Wildlife Service (WSFR—SWG Grants T‐32‐1, T‐33‐2020), USFWS Candidate Conservation Species grant, and the 2018 and 2019 Churchill Northern Studies Centre Northern Research Fund.

## CONFLICT OF INTEREST

The authors' have no conflict of interest to report. Any use of trade, firm, or product names is for descriptive purposes only and does not imply endorsement by the U.S. Government.

## Supporting information


Table S1

Table S2

## Data Availability

Movement data are available for download from MoveBank (https://www.movebank.org/cms/webapp?gwt_fragment=page=studies,path=study543061768).
