# Supplementary material for: Flyway‐scale GPS tracking reveals migratory routes and key stopover and non‐breeding locations of lesser yellowlegs
Source: Ecol Evol. 2022 Nov 9;12(11):e9495. doi: 10.1002/ece3.9495 (PMC9646513; doi:10.1002/ece3.9495)
Supplement: Supplementary file 1 — Table S1 [file ECE3-12-e9495-s002.docx]

**Table S1**. Transmission schedule for GPS tags. GPS tags were deployed in Anchorage, Alaska (ANC; 2018-2020); Eielson Air Force Base, Alaska (EIE; 2021); Kanuti National Wildlife Refuge, Alaska (KAN; 2019); Yellowknife, Northwest Territories (YKF; 2018, 2019); Churchill, Manitoba (CHU; 2019); James Bay, Ontario (JBY; 2018, 2019); and Mingan Archipelago, Quebec (MIN; 2019, 2020). Values in the dates/fix interval column indicates the scheduled transmission period (hours between each scheduled transmission). “NA” values indicate that birds from a population were not tracked in the particular year, or the number of fix interval periods for that year and population was less than 4.

|  | 2018 | | | | 2019 | | | | 2020 | | | 2021 | | | |
| --- | --- | --- | --- | --- | --- | --- | --- | --- | --- | --- | --- | --- | --- | --- | --- |
|  | Period 1 | Period 2 | Period 3 | Period 4 | Period 1 | Period 2 | Period 3 | Period 4 | Period 1 | Period 2 | Period 3 | Period 1 | Period 2 | Period 3 | Period 4 |
| Site | Dates (Fix Interval) | | | | Dates (Fix Interval) | | | | Dates (Fix Interval) | | | Dates (Fix Interval) | | | |
| ANC | 6 June to 15 July (96) | 17 July to 7 Oct (48) | 21 Oct to 10 Feb (336) | 14 Feb to 1 Jan (96) | 23 June to 18 Oct (48) | 1 Jan to 3 Jan (24) | 1 Apr to 19 May (96) | 20 May to 1 Jan (24) | 6 June to 10 July (48) | 1 Sept to 29 Apr (144) | 13 May to 1 Oct (336) | NA | NA | NA | NA |
| EIE | NA | NA | NA | NA | NA | NA | NA | NA | NA | NA | NA | 15 June to 13 July (168) | 16 July to 23 Oct (72) | 6 Nov to 7 April (336) | 9 April to 1 Jan (96) |
| KAN | NA | NA | NA | NA | 23 June to 18 Oct (48) | 1 Jan to 3 Jan (24) | 1 Apr to 19 May (96) | 20 May to 1 Jan (24) | NA | NA | NA | NA | NA | NA | NA |
| YKF | 1 July to 17 July (96) | 19 July to 7 Oct (48) | 21 Oct to 10 Feb (336) | 14 Feb to 1 Jan (96) | 1 July to 18 Oct (24) | 1 Jan to 3 Jan (24) | 1 Apr to 19 May (96) | NA | NA | NA | NA | NA | NA | NA | NA |
| CHU | NA | NA | NA | NA | 1 July to 18 Oct (24) | 1 Jan to 3 Jan (24) | 1 Apr to 1 Jan 96) | NA | NA | NA | NA | NA | NA | NA | NA |
| JBY | 1 July to 17 July (96) | 19 July to 7 Oct (48) | 21 Oct to 10 Feb (336) | 14 Feb to 1 Jan (96) | 1 Aug to 18 Nov (24) | 1 Jan to 3 Jan (24) | 1 Apr to 1 Jan (96) | NA | NA | NA | NA | NA | NA | NA | NA |
| MIN | NA | NA | NA | NA | 1 Aug to 18 Nov (24) | 1 Jan to 3 Jan (24) | 1 Apr to 1 Jan (96) | NA | 20 July to 17 Nov (96) | 1 Dec to 6 Apr (336) | 20 Apr to 1 Dec (96) | NA | NA | NA | NA |
