# Supplementary material for: Flyway‐scale GPS tracking reveals migratory routes and key stopover and non‐breeding locations of lesser yellowlegs
Source: Ecol Evol. 2022 Nov 9;12(11):e9495. doi: 10.1002/ece3.9495 (PMC9646513; doi:10.1002/ece3.9495)
Supplement: Supplementary file 2 — Table S2 [file ECE3-12-e9495-s001.docx]

**Table S2**. Summary of GPS tags deployed on Lesser Yellowlegs. Columns indicate the number of GPS tags deployed per breeding population, the number of males and females tagged per breeding population, and the number of GPS transmissions received per annual cycle of tracking.

| Site | Total Num. GPS Tags | Num. Tagged Males | Num. Tagged Females | Num. Transmissions: 2018/2019 | Num. Transmissions: 2019/2020 | Num. Transmissions: 2020/2021 | Num. Transmissions: 2021/2022 |
| --- | --- | --- | --- | --- | --- | --- | --- |
| Anchorage | 48 | 18 | 30 | 990 | 1232 | 474 | NA |
| Eielson AFB | 5 | undetermined | undetermined | NA | NA | NA | 150 |
| Kanuti NWR | 10 | 4 | 6 | NA | 455 | NA | NA |
| Yellowknife | 11 | 7 | 4 | 50 | 623 | NA | NA |
| Churchill | 20 | 11 | 9 | NA | 1173 | NA | NA |
| James Bay | 9 | 5 | 4 | 193 | 85 | NA | NA |
| Mingan | 15 | 7 | 7 (1 unknown sex) | NA | 88 | 325 | NA |
